# Supplementary figures and images for: Identification of QTLs for high grain yield and component traits in new plant types of rice
Source: PLoS One. 2020 Jul 16;15(7):e0227785. doi: 10.1371/journal.pone.0227785 (PMC7365460; doi:10.1371/journal.pone.0227785)

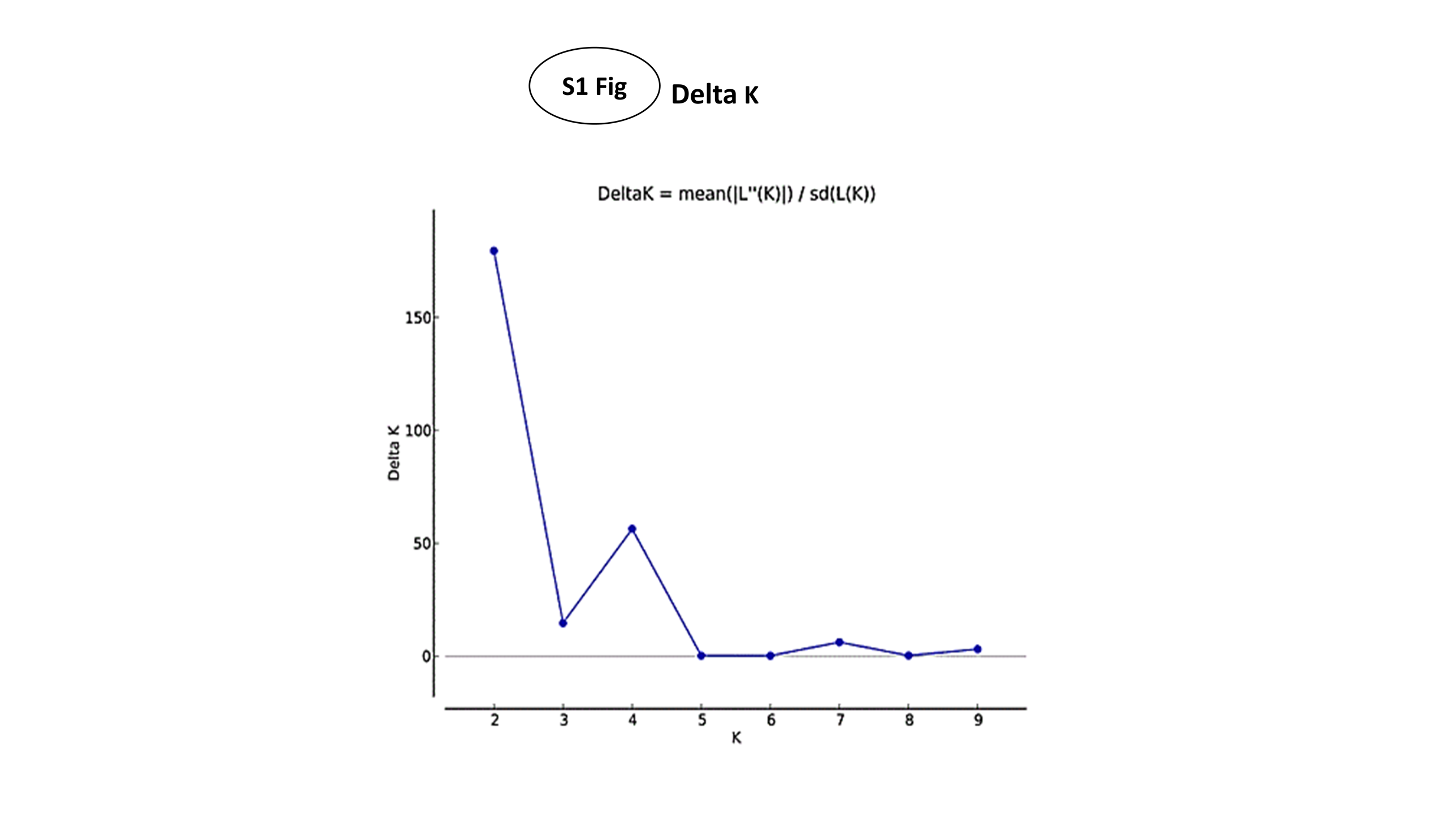

Supplement: S1 Fig — (TIF) [file pone.0227785.s001.tif]

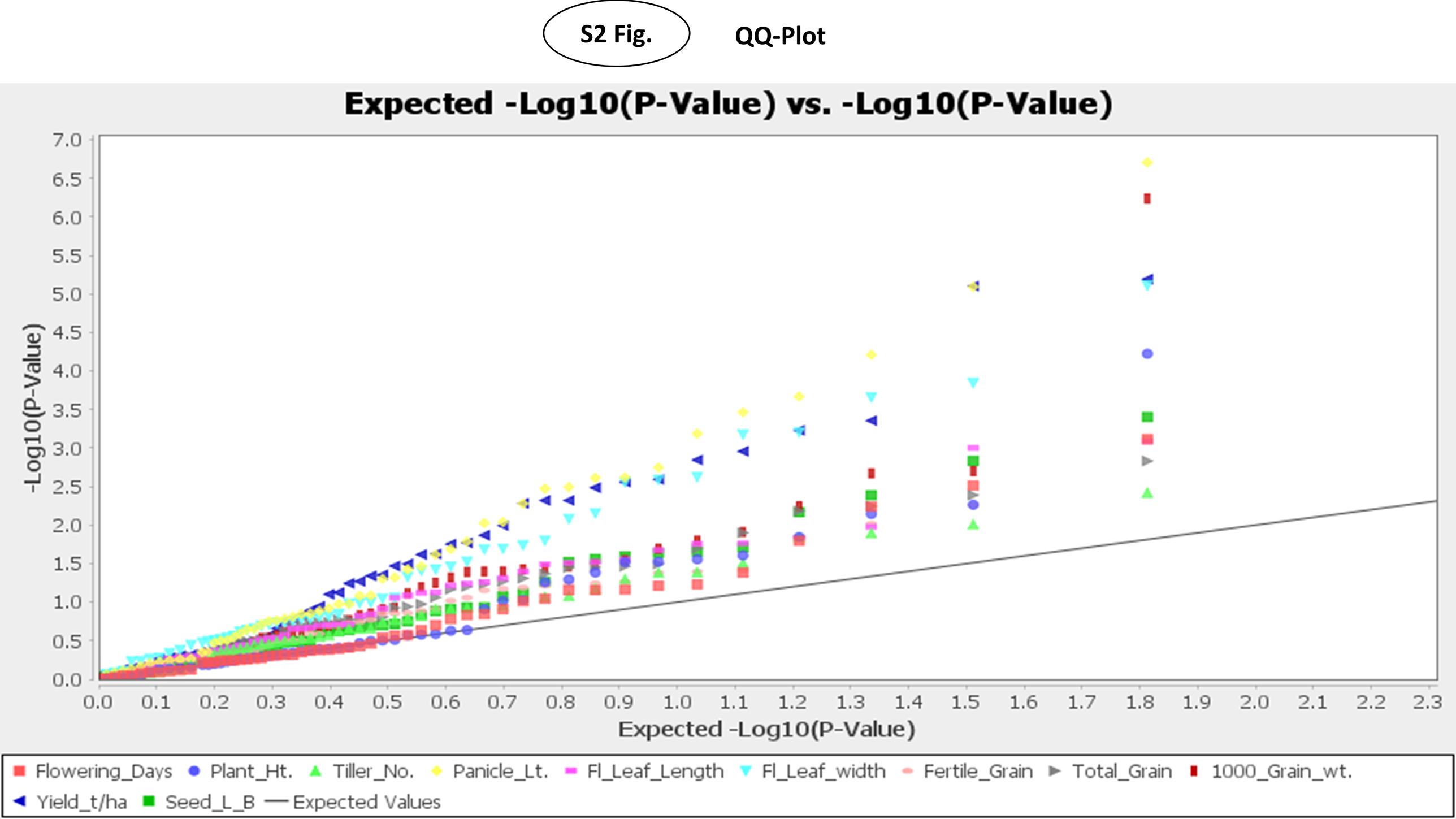

Supplement: S2 Fig — (TIF) [file pone.0227785.s002.tif]

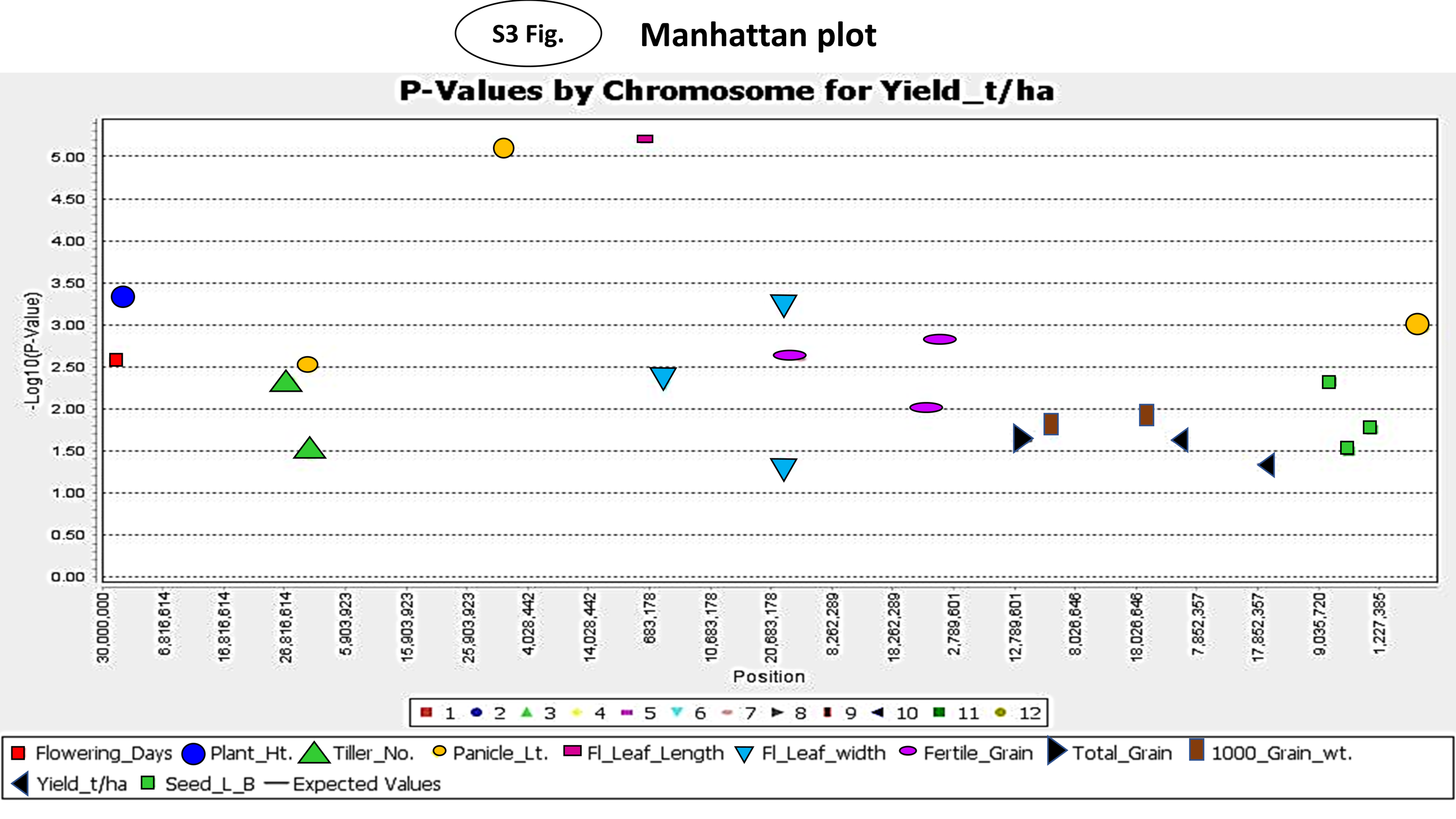

Supplement: S3 Fig — (TIF) [file pone.0227785.s003.tif]

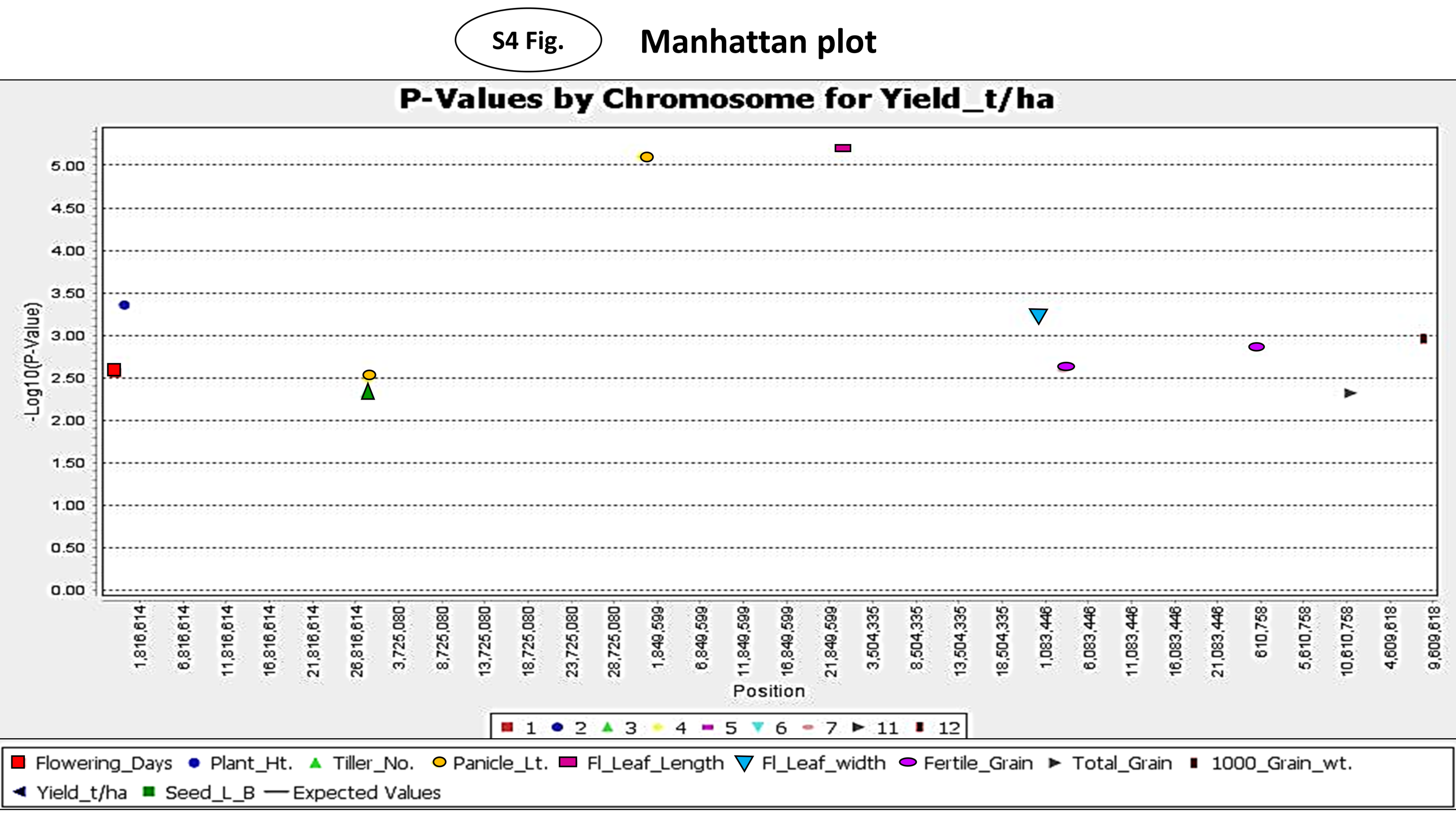

Supplement: S4 Fig — (TIF) [file pone.0227785.s004.tif]
