# Supplementary material for: Identification of QTLs for high grain yield and component traits in new plant types of rice
Source: PLoS One. 2020 Jul 16;15(7):e0227785. doi: 10.1371/journal.pone.0227785 (PMC7365460; doi:10.1371/journal.pone.0227785)
Supplement: S1 Table — (DOCX) [file pone.0227785.s005.docx]

**S1 Table.** **List of 48 New plant types (NPT) and 12 popular DUS reference genotypes used in association mapping analysis.**

| **Code no.** | **Genotype code** | **Designation** | **Code no.** | **Genotype code** | **Designation** | **Code no.** | **Reference Genotypes (DUS)** | |
| --- | --- | --- | --- | --- | --- | --- | --- | --- |
| **1** | **N-366** | **IR-72058-26-3-2-3-6-6-1** | **25** | **N-110** | **IR-73930-41-5-3-1-3** | 49 | Naveen | **Indica** |
| **2** | **N-361** | **IR-73930-41-5-3-1-2** | **26** | **N-353** | **IR-73930-313-2-2-1** | 50 | Lalat | **Indica** |
| **3** | **N-336** | **IR-73933-8-2-2-3-5-10** | **27** | **N-373** | **IR-73931-40-1-2-3-2-2-1** | 51 | Sambahmasuri | **Indica** |
| **4** | **N-369** | **CR-3727-12-1** | **28** | **N-318** | **IR-73931-40-1-2-3-2-4-1** | 52 | Swarna | **Indica** |
| **5** | **N-89** | **IR-75163-45-2-5-3-5-1-1** | **29** | **N-129** | **CR-3723-3-1-1** | 53 | IR-64 | **Indica** |
| **6** | **N-49** | **IR-73995-13-1-3-2-4-1** | **30** | **R-260** | **CR-3855-2-1** | 54 | MTU 1010 | **Indica** |
| **7** | **R-255** | **IR-82489-7-2-2-2-1** | **31** | **N-312** | **IR-73933-106-2-1-2-1-1** | 55 | [Nipponbare](https://www.google.co.in/search?q=rice+nipponbare&spell=1&sa=X&ved=0ahUKEwi22Pbg37TSAhUIrJQKHXWYCNoQvwUIGCgA) | **Temp. Japonica** |
| **8** | **N-41** | **IR-72158-26-3-2-3-3** | **32** | **N-316** | **IR-72158-26-3-2-3-3-2** | 56 | C-105A51 | **Temp. Japonica** |
| **9** | **R-261** | **IR-77700-84-2-2-2-1** | **33** | **N-65** | **IR-73930-31-3-2-2-2** | 57 | AC41009 (Peta) | **Temp. Japonica** |
| **10** | **N-334** | **IR-73930-31-3-2-2-2-2** | **34** | **N-352** | **IR-73930-313-2-2-2-2** | 58 | Azucena | **Trop. Japonica** |
| **11** | **N-2** | **IR-71700-247-5-3-2-1-2-2-1** | **35** | **N-100** | **IR-73930-313-2-2-7** | 59 | Curinga | **Trop. Japonica** |
| **12** | **N-8** | **IR-72158-68-6-3-3-1** | **36** | **N-370** | **IR-71700-5-3-2-2-1** | 60 | WC-8 | **Trop. Japonica** |
| **13** | **N-358** | **IR-73930-41-5-3-1-3-1** | **37** | **N-337** | **IR-73933-8-2-2-3-5** |  |  |  |
| **14** | **N-333** | **CR-3728-2** | **38** | **N-309** | **IR-71701-28-1-4-1-1** |  |  |  |
| **15** | **N-323** | **IR-71700-247-5-3-2-1-11-1-3** | **39** | **N-374** | **IR-73931-40-1-2-3-2-2** |  |  |  |
| **16** | **N-65-1** | **IR-73930-31-3-2-2-2-1** | **40** | **N-91** | **IR-75163-45-2-5-3-7** |  |  |  |
| **17** | **N-34** | **IR-78629-57-3-3-9-5** | **41** | **N-310** | **IR-72158-68-3-3** |  |  |  |
| **18** | **N-306** | **IR-72158-68-6-3-1-1** | **42** | **N-66** | **IR-73930-31-3-2-2-2-2** |  |  |  |
| **19** | **N-3** | **IR-71700-5-3-2-1-3** | **43** | **N-302** | **IR-71700-247-5-3-2-1-3-1** |  |  |  |
| **20** | **N-5** | **IR-72158-154-3-2-1-1-2-1** | **44** | **N-320** | **IR-71700-247-5-3-2-1-5** |  |  |  |
| **21** | **N-17** | **IR-71701-28-1-4-1** | **45** | **N-39** | **IR-72158-26-3-2-3-1** |  |  |  |
| **22** | **N-26** | **IR73931-40-9-2-3-2-1** | **46** | **N-135** | **IR-74714-141-3-3-2-4-3** |  |  |  |
| **23** | **N-43** | **IR73933-8-2-2-3-1** | **47** | **N-331** | **CR-3721-11-1** |  |  |  |
| **24** | **N-76** | **IR 73896-51-2-1-3-1-1** | **48** | **N-79** | **IR-73896-51-2-1-3-4** |  |  |  |
