# Supplementary material for: Identification of QTLs for high grain yield and component traits in new plant types of rice
Source: PLoS One. 2020 Jul 16;15(7):e0227785. doi: 10.1371/journal.pone.0227785 (PMC7365460; doi:10.1371/journal.pone.0227785)
Supplement: S2 Table — (DOCX) [file pone.0227785.s006.docx]

**S2 Table. Grain yield performance of 20 best varieties and standard checks under irrigated condition.**

| **Variety** | **2011 *Yr.*** | **2012 *Yr.*** | **2013 *Yr.*** | **2014 *Yr.*** | **Average** | **Standard Deviation** | **Standard Error** |
| --- | --- | --- | --- | --- | --- | --- | --- |
| N_129 | 9.12 | 9.89 | 6.59 | 9.60 | **8.80** | **1.51** | **0.75** |
| N_8 | 6.63 | 6.88 | 6.34 | 8.86 | **7.18** | **1.15** | **0.57** |
| R_255 | 7.30 | 6.72 | 5.85 | 8.72 | **7.14** | **1.21** | **0.60** |
| N_370 | 7.39 | 8.78 | 6.07 | 8.20 | **7.61** | **1.18** | **0.59** |
| R_261 | 8.04 | 6.05 | 6.05 | 8.06 | **7.05** | **1.16** | **0.58** |
| N_17 | 6.61 | 6.90 | 5.54 | 8.02 | **6.77** | **1.02** | **0.51** |
| N_5 | 8.02 | 7.22 | 5.44 | 7.95 | **7.16** | **1.20** | **0.60** |
| R_260 | 7.02 | 7.31 | 6.27 | 7.35 | **6.99** | **0.50** | **0.25** |
| N_310 | 6.73 | 7.04 | 5.58 | 7.29 | **6.66** | **0.75** | **0.38** |
| N_79 | 7.21 | 7.27 | 5.90 | 7.27 | **6.91** | **0.68** | **0.34** |
| N_2 | 6.35 | 6.51 | 6.29 | 7.23 | **6.59** | **0.43** | **0.22** |
| N_89 | 7.04 | 7.28 | 6.27 | 7.21 | **6.95** | **0.47** | **0.23** |
| N_337 | 7.90 | 7.07 | 5.36 | 7.07 | **6.85** | **1.07** | **0.53** |
| N_135 | 6.62 | 7.22 | 5.81 | 7.06 | **6.68** | **0.63** | **0.32** |
| N_323 | 7.21 | 7.28 | 5.79 | 7.02 | **6.82** | **0.70** | **0.35** |
| N_76 | 7.15 | 6.95 | 5.43 | 7.00 | **6.63** | **0.81** | **0.40** |
| N302 | 6.98 | 6.32 | 5.63 | 6.14 | **6.27** | **0.56** | **0.28** |
| N306 | 6.03 | 6.00 | 4.59 | 6.96 | **5.89** | **0.98** | **0.49** |
| N333 | 5.67 | 5.53 | 4.77 | 6.66 | **5.65** | **0.78** | **0.39** |
| N39 | 5.77 | 5.58 | 4.35 | 6.10 | **5.45** | **0.76** | **0.38** |
| **MTU_1010** | 5.33 | 5.31 | 4.11 | 5.20 | **4.99** | **0.59** | **0.30** |
| **IR_64** | 5.37 | 5.25 | 3.71 | 4.89 | **4.80** | **0.76** | **0.38** |
| **Standard Deviation** | **0.93** | **1.07** | **0.78** | **1.11** | **0.88** | **0.29** | **0.15** |
| **Standard Error** | **0.22** | **0.25** | **0.18** | **0.26** | **0.21** | **0.07** | **0.03** |
