# Supplementary material for: Identification of QTLs for high grain yield and component traits in new plant types of rice
Source: PLoS One. 2020 Jul 16;15(7):e0227785. doi: 10.1371/journal.pone.0227785 (PMC7365460; doi:10.1371/journal.pone.0227785)
Supplement: S3 Table — (DOCX) [file pone.0227785.s007.docx]

**S3 Table. Correlation matrix of grain yield and their association with 10 yield-related traits**

| **Variables** | **DFF** | **PH** | **TL** | **PL** | **FLL** | **FLW** | **FG** | **TG** | **TGW** | **SLBR** | **YLD** |
| --- | --- | --- | --- | --- | --- | --- | --- | --- | --- | --- | --- |
| **DFF** | **1** | **0.606** | 0.249 | **0.516** | **0.516** | **0.457** | **0.604** | **0.557** | -0.092 | 0.119 | **0.480** |
| **PH** | **0.606** | **1** | -0.017 | **0.669** | **0.731** | **0.386** | **0.367** | **0.359** | 0.037 | 0.089 | **0.418** |
| **TL** | 0.249 | -0.017 | **1** | **0.317** | 0.154 | **0.352** | **0.273** | **0.290** | 0.026 | 0.152 | **0.559** |
| **PL** | **0.516** | **0.669** | **0.317** | **1** | **0.747** | **0.658** | **0.473** | **0.504** | **0.312** | **0.335** | **0.729** |
| **FLL** | **0.516** | **0.731** | 0.154 | **0.747** | **1** | **0.556** | **0.354** | **0.419** | 0.214 | **0.351** | **0.552** |
| **FLW** | **0.457** | **0.386** | **0.352** | **0.658** | **0.556** | **1** | **0.510** | **0.577** | **0.444** | 0.229 | **0.525** |
| **FG** | **0.604** | **0.367** | **0.273** | **0.473** | **0.354** | **0.510** | **1** | **0.944** | 0.105 | 0.090 | **0.383** |
| **TG** | **0.557** | **0.359** | **0.290** | **0.504** | **0.419** | **0.577** | **0.944** | **1** | 0.180 | 0.133 | **0.359** |
| **TGW** | -0.092 | 0.037 | 0.026 | **0.312** | 0.214 | **0.444** | 0.105 | 0.180 | **1** | 0.065 | 0.103 |
| **SLBR** | 0.119 | 0.089 | 0.152 | **0.335** | **0.351** | 0.229 | 0.090 | 0.133 | 0.065 | **1** | 0.170 |
| **YLD** | **0.480** | **0.418** | **0.559** | **0.729** | **0.552** | **0.525** | **0.383** | **0.359** | 0.103 | 0.170 | **1** |

*Note: Values in bold are different from 0 with a significance level alpha=0.05*
