# Supplementary material for: Identification of QTLs for high grain yield and component traits in new plant types of rice
Source: PLoS One. 2020 Jul 16;15(7):e0227785. doi: 10.1371/journal.pone.0227785 (PMC7365460; doi:10.1371/journal.pone.0227785)
Supplement: S4 Table — (DOCX) [file pone.0227785.s008.docx]

**S4 Table. Calculation of Standardized coefficients**

| **Source** | **Value** | **Standard error** | **t** | **Pr > \|t\|** | **Lower bound (95%)** | **Upper bound (95%)** |
| --- | --- | --- | --- | --- | --- | --- |
| **Flowering Days (DFF)** | 0.089 | 0.120 | 0.746 | 0.460 | -0.151 | 0.330 |
| **Plant Ht. (PH)** | -0.211 | 0.145 | -1.453 | 0.152 | -0.503 | 0.081 |
| **Tiller No. (TL)** | 0.336 | 0.090 | 3.731 | 0.000 | 0.155 | 0.517 |
| **Panicle Lt. (PL)** | 0.649 | 0.147 | 4.410 | < 0.0001 | 0.353 | 0.945 |
| **Seed L B (SLBR)** | -0.147 | 0.087 | -1.690 | 0.097 | -0.322 | 0.028 |
| **Flag Leaf Length (FLL)** | 0.230 | 0.143 | 1.607 | 0.115 | -0.058 | 0.517 |
| **Flag Leaf width (FLW)** | 0.075 | 0.125 | 0.598 | 0.553 | -0.176 | 0.325 |
| **Fertile Grain (FG)** | 0.406 | 0.258 | 1.574 | 0.122 | -0.112 | 0.924 |
| **Total Grain (TG)** | -0.525 | 0.258 | -2.038 | 0.047 | -1.043 | -0.007 |
| **1000 Grain wt. (TGW)** | -0.116 | 0.095 | -1.219 | 0.229 | -0.307 | 0.075 |
