# Supplementary material for: Identification of QTLs for high grain yield and component traits in new plant types of rice
Source: PLoS One. 2020 Jul 16;15(7):e0227785. doi: 10.1371/journal.pone.0227785 (PMC7365460; doi:10.1371/journal.pone.0227785)
Supplement: S5 Table — (DOCX) [file pone.0227785.s009.docx]

**S5 Table. Distribution pattern of genotypes in Principal Component Analysis (PCA) and Biplot by using morphological-physiological data.**

| **Quadrant** | **Color Circle** | **Genotypes** | **Predominant Traits Controlling Yield** |
| --- | --- | --- | --- |
| **I** | **Green** | N_129, N_370, N_302, R_261, N_17, N_309, N_100, N_353, N_310, N_79, N_89, N_337, N_334, N_135, N_49, N_110, N_374, N_333, N_312, N_316, N_2, N_66, N_316, N_8 | **YLD, TL, FLL, FLW, TGW, SLBR, PL** |
| **II and III** | **Red** | R_260, N_318, Lalat, MTU1010, IR_64, N_65, Azucena,N_43, Naveen, Samba Masuri | **----** |
| **II and III** | **Blue** | Curinga, C_105A51, Peta, Nipponbare | **----** |
| **IV** | **Pink** | N_369, N_320, N_353, N_76, N_34, N_352, N_26, N_331, N_323, wc_8, N_331, R_255, N_358, N_5, N_306, N_3, | **TG, FG, PH, DFF** |
