# Supplementary material for: Identification of QTLs for high grain yield and component traits in new plant types of rice
Source: PLoS One. 2020 Jul 16;15(7):e0227785. doi: 10.1371/journal.pone.0227785 (PMC7365460; doi:10.1371/journal.pone.0227785)
Supplement: S6 Table — (DOCX) [file pone.0227785.s010.docx]

**S6 Table.** **Molecular diversity among 60 rice genotypes based on the alleles amplified by 66 polymorphic SSR markers.**

| **Sr.NO** | **PRIMER** | **Chrm#** | **TA** | **PA** | **UA** | **RA** | **Major Allele Frequency** | **Gene Diversity** | **PIC** | **Ne** | **Ho** | **He** | **I** |
| --- | --- | --- | --- | --- | --- | --- | --- | --- | --- | --- | --- | --- | --- |
| 1 | RM 212 | 1 | 3 | 3 | 0 | 0 | 0.6833 | 0.4817 | 0.83 | 1.929 | 0.514 | 0.482 | 0.840 |
| 2 | RM 220 | 1 | 2 | 2 | 0 | 0 | 0.5667 | 0.4911 | 0.74 | 1.973 | 0.503 | 0.493 | 0.686 |
| 3 | RM 297 | 1 | 2 | 2 | 0 | 0 | 0.8833 | 0.2061 | 0.60 | 1.260 | 0.792 | 0.206 | 0.360 |
| 4 | RM 495 | 1 | 2 | 2 | 0 | 0 | 0.7250 | 0.3988 | 0.69 | 1.663 | 0.598 | 0.399 | 0.588 |
| 5 | RM 1282 | 1 | 2 | 2 | 0 | 0 | 0.6333 | 0.4644 | 0.72 | 1.867 | 0.532 | 0.464 | 0.657 |
| 6 | RM263 | 2 | 2 | 2 | 0 | 0 | 0.7167 | 0.4061 | 0.70 | 1.704 | 0.583 | 0.413 | 0.604 |
| 7 | RM 521 | 2 | 3 | 3 | 0 | 0 | 0.4833 | 0.6294 | 0.87 | 2.790 | 0.353 | 0.642 | 1.062 |
| 8 | RM 7144 | 2 | 2 | 2 | 0 | 0 | 0.5167 | 0.4994 | 0.73 | 1.991 | 0.498 | 0.498 | 0.691 |
| 9 | RM 154 | 2 | 4 | 4 | 0 | 0 | 0.3500 | 0.7211 | 0.90 | 3.586 | 0.273 | 0.721 | 1.327 |
| 10 | RM 324 | 2 | 2 | 2 | 0 | 0 | 0.6750 | 0.4388 | 0.69 | 1.782 | 0.558 | 0.439 | 0.631 |
| 11 | RM 16 | 3 | 3 | 3 | 0 | 0 | 0.6583 | 0.4829 | 0.81 | 1.934 | 0.513 | 0.483 | 0.798 |
| 12 | RM 7 | 3 | 2 | 2 | 0 | 0 | 0.8167 | 0.2994 | 0.65 | 1.427 | 0.698 | 0.299 | 0.476 |
| 13 | RM 168 | 3 | 3 | 3 | 0 | 1 | 0.7333 | 0.4067 | 0.80 | 1.685 | 0.590 | 0.407 | 0.680 |
| 14 | RM 6266 | 3 | 2 | 2 | 1 | 1 | 0.9833 | 0.0328 | 0.52 | 1.034 | 0.967 | 0.033 | 0.085 |
| 15 | RM 520 | 3 | 2 | 2 | 0 | 0 | 0.8167 | 0.2994 | 0.65 | 1.427 | 0.698 | 0.299 | 0.476 |
| 16 | RM 1256 | 3 | 2 | 2 | 0 | 0 | 0.7167 | 0.4061 | 0.70 | 1.684 | 0.591 | 0.406 | 0.596 |
| 17 | RM 489 | 3 | 2 | 2 | 1 | 1 | 0.9833 | 0.0328 | 0.52 | 1.034 | 0.967 | 0.033 | 0.085 |
| 18 | RM 6569 | 3 | 2 | 2 | 0 | 0 | 0.9167 | 0.1528 | 0.58 | 1.180 | 0.846 | 0.153 | 0.287 |
| 19 | RM 551 | 4 | 2 | 2 | 0 | 0 | 0.5167 | 0.4994 | 0.75 | 1.998 | 0.496 | 0.499 | 0.693 |
| 20 | RM 470 | 4 | 2 | 2 | 0 | 0 | 0.7500 | 0.3750 | 0.69 | 1.600 | 0.622 | 0.375 | 0.562 |
| 21 | RM 3276 | 4 | 2 | 2 | 0 | 1 | 0.9500 | 0.0950 | 0.55 | 1.105 | 0.904 | 0.095 | 0.199 |
| 22 | RM 5709 | 4 | 4 | 4 | 0 | 2 | 0.7083 | 0.4588 | 0.86 | 1.848 | 0.537 | 0.459 | 0.871 |
| 23 | RM 164 | 4 | 3 | 3 | 0 | 0 | 0.6583 | 0.4929 | 0.83 | 1.972 | 0.503 | 0.493 | 0.832 |
| 24 | RM 334 | 4 | 2 | 2 | 0 | 0 | 0.5833 | 0.4861 | 0.73 | 1.965 | 0.505 | 0.491 | 0.684 |
| 25 | RM 413 | 5 | 2 | 2 | 0 | 0 | 0.7667 | 0.3578 | 0.68 | 1.557 | 0.639 | 0.358 | 0.543 |
| 26 | RM 18004 | 5 | 2 | 2 | 0 | 0 | 0.9333 | 0.1244 | 0.56 | 1.142 | 0.875 | 0.124 | 0.245 |
| 27 | RM 5575 | 5 | 2 | 2 | 0 | 0 | 0.8250 | 0.2888 | 0.52 | 1.406 | 0.709 | 0.289 | 0.464 |
| 28 | RM 204 | 5 | 4 | 4 | 0 | 0 | 0.5250 | 0.6282 | 0.92 | 2.690 | 0.367 | 0.628 | 1.151 |
| 29 | RM 439 | 5 | 3 | 3 | 0 | 0 | 0.7667 | 0.3844 | 0.79 | 1.625 | 0.612 | 0.384 | 0.703 |
| 30 | RM 528 | 6 | 2 | 2 | 0 | 1 | 0.9500 | 0.0950 | 0.55 | 1.105 | 0.904 | 0.095 | 0.199 |
| 31 | RM 3827 | 6 | 2 | 2 | 0 | 0 | 0.8833 | 0.2061 | 0.60 | 1.260 | 0.792 | 0.206 | 0.360 |
| 32 | RM 20285 | 6 | 2 | 2 | 0 | 0 | 0.7167 | 0.4061 | 0.70 | 1.684 | 0.591 | 0.406 | 0.596 |
| 33 | RM 70 | 6 | 4 | 4 | 0 | 1 | 0.4333 | 0.6683 | 0.82 | 3.015 | 0.326 | 0.668 | 1.180 |
| 34 | RM 234 | 6 | 2 | 2 | 0 | 0 | 0.7000 | 0.4200 | 0.69 | 1.724 | 0.577 | 0.420 | 0.611 |
| 35 | RM 1132 | 7 | 4 | 4 | 0 | 0 | 0.3250 | 0.7321 | 0.91 | 3.733 | 0.262 | 0.732 | 1.347 |
| 36 | RM 3555 | 7 | 2 | 2 | 0 | 0 | 0.8667 | 0.2311 | 0.62 | 1.301 | 0.767 | 0.231 | 0.393 |
| 37 | RM 5711 | 7 | 3 | 3 | 0 | 0 | 0.4500 | 0.5961 | 0.87 | 2.476 | 0.399 | 0.596 | 0.972 |
| 38 | RM 25 | 7 | 3 | 3 | 0 | 0 | 0.4667 | 0.6365 | 0.88 | 2.751 | 0.358 | 0.637 | 1.054 |
| 39 | RM 447 | 7 | 2 | 2 | 0 | 0 | 0.6333 | 0.4644 | 0.72 | 1.867 | 0.532 | 0.464 | 0.657 |
| 40 | RM 152 | 8 | 2 | 2 | 0 | 0 | 0.5667 | 0.4911 | 0.67 | 1.965 | 0.505 | 0.491 | 0.684 |
| 41 | RM 22899 | 8 | 2 | 2 | 0 | 0 | 0.7417 | 0.3832 | 0.65 | 1.621 | 0.614 | 0.383 | 0.571 |
| 42 | RM 3491 | 8 | 2 | 2 | 0 | 0 | 0.7583 | 0.3665 | 0.67 | 1.579 | 0.630 | 0.367 | 0.553 |
| 43 | RM 201 | 8 | 2 | 2 | 0 | 0 | 0.7833 | 0.3394 | 0.65 | 1.514 | 0.658 | 0.339 | 0.523 |
| 44 | RM 219 | 8 | 2 | 2 | 0 | 0 | 0.7167 | 0.4061 | 0.70 | 1.684 | 0.591 | 0.406 | 0.596 |
| 45 | RM 285 | 9 | 3 | 3 | 0 | 0 | 0.6417 | 0.4907 | 0.81 | 1.920 | 0.517 | 0.479 | 0.784 |
| 46 | RM 5688 | 9 | 3 | 3 | 0 | 0 | 0.5833 | 0.5617 | 0.85 | 2.281 | 0.434 | 0.562 | 0.940 |
| 47 | RM 6920 | 9 | 2 | 2 | 0 | 0 | 0.7333 | 0.3911 | 0.68 | 1.642 | 0.606 | 0.391 | 0.580 |
| 48 | RM 274 | 9 | 2 | 2 | 0 | 0 | 0.8500 | 0.2550 | 0.61 | 1.342 | 0.743 | 0.255 | 0.423 |
| 49 | RM 5348 | 9 | 2 | 2 | 0 | 0 | 0.8583 | 0.2432 | 0.60 | 1.342 | 0.743 | 0.255 | 0.423 |
| 50 | RM 171 | 10 | 2 | 2 | 0 | 0 | 0.8167 | 0.2994 | 0.65 | 1.427 | 0.698 | 0.299 | 0.476 |
| 51 | RM 222 | 10 | 2 | 2 | 0 | 0 | 0.7583 | 0.3665 | 0.67 | 1.579 | 0.630 | 0.367 | 0.553 |
| 52 | RM 333 | 10 | 2 | 2 | 0 | 0 | 0.8833 | 0.2061 | 0.60 | 1.260 | 0.792 | 0.206 | 0.360 |
| 53 | RM 6100 | 10 | 2 | 2 | 0 | 0 | 0.7167 | 0.4061 | 0.70 | 1.684 | 0.591 | 0.406 | 0.596 |
| 54 | RM 286 | 10 | 2 | 2 | 0 | 0 | 0.8500 | 0.2550 | 0.63 | 1.342 | 0.743 | 0.255 | 0.423 |
| 55 | RM 21 | 10 | 2 | 2 | 0 | 0 | 0.6667 | 0.4444 | 0.69 | 1.800 | 0.552 | 0.444 | 0.637 |
| 56 | RM 206 | 11 | 3 | 3 | 0 | 0 | 0.6917 | 0.4740 | 0.82 | 1.901 | 0.522 | 0.474 | 0.831 |
| 57 | RM 209 | 11 | 2 | 2 | 0 | 0 | 0.7667 | 0.3578 | 0.68 | 1.557 | 0.639 | 0.358 | 0.543 |
| 58 | RM 287 | 11 | 2 | 2 | 0 | 0 | 0.5167 | 0.4994 | 0.75 | 1.998 | 0.496 | 0.499 | 0.693 |
| 59 | RM 7226 | 11 | 2 | 2 | 0 | 0 | 0.6917 | 0.4265 | 0.69 | 1.744 | 0.570 | 0.427 | 0.618 |
| 60 | RM 26499 | 11 | 2 | 2 | 0 | 0 | 0.7250 | 0.3988 | 0.64 | 1.663 | 0.598 | 0.399 | 0.588 |
| 61 | RM 202 | 11 | 3 | 3 | 0 | 0 | 0.3898 | 0.6583 | 0.86 | 2.926 | 0.336 | 0.658 | 1.086 |
| 62 | RM 17 | 11 | 2 | 2 | 0 | 0 | 0.6583 | 0.4499 | 0.68 | 1.818 | 0.546 | 0.450 | 0.642 |
| 63 | RM 19 | 11 | 2 | 2 | 0 | 0 | 0.7667 | 0.3578 | 0.64 | 1.557 | 0.639 | 0.358 | 0.543 |
| 64 | RM 1103 | 12 | 2 | 2 | 0 | 0 | 0.8583 | 0.2432 | 0.61 | 1.321 | 0.755 | 0.243 | 0.408 |
| 65 | RM 3331 | 12 | 2 | 2 | 0 | 0 | 0.7750 | 0.3488 | 0.65 | 1.579 | 0.630 | 0.367 | 0.553 |
| 66 | RM 1261 | 12 | 2 | 2 | 0 | 0 | 0.7417 | 0.3832 | 0.68 | 1.621 | 0.614 | 0.383 | 0.571 |
|  |  |  | **TA** | **PA** | **UA** | **RA** | **Major Allele Frequency** | **Gene Diversity** | **PIC** | Ne | Ho | He | I |
|  |  | Total | **154** | **154** | **2** | **8** | **46.77316384** | **25.99981563** | **46.46122** | **117.4403** | **39.739** | **26.0416** | **41.4437** |
|  |  | Avg. | 2.33 | 2.33 | 0.03 | 0.12 | 0.71 | 0.39 | 0.70 | 1.78 | 0.60 | 0.39 | 0.63 |
|  |  | % |  | 100.00 | 1.30 | 5.19 | 30.37 | 16.88 | 30.17 | 76.26 | 25.80 | 16.91 | 26.91 |

TA = Total number of alleles, PA = Polymorphic alleles, UA= Unique alleles, RA=Rare alleles, PIC=Polymorphism information contents, Ne: Number of effective alleles, Ho: Expected homozygosity, He: Nei's genetic diversity and I: Shannon's information/diversity index
