# Supplementary material for: Identification of QTLs for high grain yield and component traits in new plant types of rice
Source: PLoS One. 2020 Jul 16;15(7):e0227785. doi: 10.1371/journal.pone.0227785 (PMC7365460; doi:10.1371/journal.pone.0227785)
Supplement: S7 Table — (DOCX) [file pone.0227785.s011.docx]

**S7 Table. Correlation between PIC and different types of alleles.**

| **Correlation with** | **Correlation** | **t-Value** | **Degree of Freedom** | **One tail Probability** | **Two tail Probability** |
| --- | --- | --- | --- | --- | --- |
| r(PIC_TA) | 0.8186 | 11.4018 | 64 | 0 | 0 |
| r(PIC_RA) | **-0.0625** | -0.5002 | 64 | 0.69067505 | 0.6186499 |
| r(PIC_LFA) | 0.4171 | 3.6717 | 64 | 0.00024673 | 0.00049346 |
| r(PIC_HFA) | 0.4628 | 4.1765 | 64 | 0.00004567 | 0.00009134 |
